# Microfluidic Encapsulation of Single Cells by Alginate Microgels Using a Trigger-Gellified Strategy

**DOI:** 10.3389/fbioe.2020.583065

**Published:** 2020-10-14

**Authors:** Fei Shao, Lei Yu, Yang Zhang, Chuanfeng An, Haoyue Zhang, Yujie Zhang, Yi Xiong, Huanan Wang

**Affiliations:** ^1^Key State Laboratory of Fine Chemicals, School of Bioengineering, Dalian University of Technology, Dalian, China; ^2^Laboratory of Regenerative Biomaterials, Department of Biomedical Engineering, Health Science Center, Shenzhen University, Shenzhen, China

**Keywords:** microfluidics, alginate microgels, cell encapsulation, calcium complexes, on-chip gelation, biocomatability

## Abstract

Microfluidics-based alginate microgels have shown great potential to encapsulate cells in a high-throughput and controllable manner. However, cell viability and biological functions are substantially compromised due to the harsh conditions for gelation, which remains a major challenge for cell encapsulation. Herein, we presented an efficient and biocompatible method by on-chip triggered gelation to generate microfluidic alginate microgels for single-cell encapsulation. Two calcium complexes of calcium–ethylenediaminetetraacetic acid (Ca-EDTA) and calcium–nitrilotriacetic (Ca-NTA) as crosslinkers for triggered gelation of alginate were compared and investigated for feasible application. By triggered release of Ca^2+^ ions from the calcium complex via adding acetic acid in the oil phase, the alginate precursor in the aqueous droplets can be crosslinked to form alginate microgels. Although using Ca-EDTA and Ca-NTA both achieved on-chip gelation, Ca-NTA led to significantly higher cell viability since the dissociation of Ca^2+^ ions from Ca-NTA can be obtained using less concentration of acid compared to Ca-EDTA. We further demonstrated the functionality of encapsulated mesenchymal stem cells (MSCs) in alginate microgels prepared using Ca-NTA, as evidenced by the osteogenesis of encapsulated MSCs upon inductive culture. In summary, our study provided a biocompatible strategy to prepare alginate microgels for single-cell encapsulation which can be further used for applications in tissue engineering and cell therapies.

## Introduction

Constructing cell carriers has become a powerful way to address biomedical questions, such as screening and delivery of drugs (Holloway et al., 2014; Yeung et al., 2016; Kang et al., 2020), cell therapy (Lim and Sun, 1980; Burdick et al., 2016), and tissue regeneration (Caiazzo et al., 2016; An et al., 2020). Hydrogels containing hydrophilic polymeric networks are widely used as matrix materials to construct cell carriers because it resembles a natural extracellular matrix (ECM) and can be further functionalized to offer physiologically relevant environmental cues (Borenstein et al., 2007; Mao et al., 2017). However, conventional bulk hydrogels have limited diffusion efficiency, which may hamper efficient nutrition and oxygen transfer, thus resulting in restricted intercellular communication and inaccurate control of cell behavior. With this aim, micrometer-sized hydrogels (microgels) were extensively studied as cell vehicles. Moreover, cell transplantation in uniform microgels can offer administration by injection in a minimally invasive manner, which enables the promotion of cell viability and leveraged therapeutic activities of encapsulated cells (Velasco et al., 2012; Wan, 2012). Besides, due to the tight microenvironment control of cells, cells encapsulated in microgels can be served as a tissue engineering module for constructing large tissues and organs.

To produce such microgel modules containing cells in a high-throughput manner, several microfabrication techniques such as micro-molding, photolithography, and flow lithography have been developed (Selimovic et al., 2012; Yun et al., 2013; Kang et al., 2014). However, most of them are limited for further application due to relatively low throughput and impaired cell viability and activity during processing (Franco et al., 2011; Choi et al., 2016). To this end, microfluidic technology has been considered as a practical method to obtain microgels given its controllable and biocompatible generation process.

Alginate microgels have been mostly used for cell encapsulation (Moyer et al., 2010; Hu et al., 2017), drug delivery (Somo et al., 2017; Uyen et al., 2019), and tissue engineering (Goh et al., 2012; Kumar Meena et al., 2019) due to its biocompatibility and ease of gelation. Lim and Sun (1980) firstly used alginate microgels to encapsulate islet cells to escape from the immune clearance and prolong retention time after implantation. It was also used as a vesicle to deliver MSCs’ (Mao et al., 2017; Campiglio et al., 2020), chondrocytes (Markstedt et al., 2015; An et al., 2020), and murine pre-adipocyte cells (Mao et al., 2017) for tissue regeneration. Regarding the microfluidic fabrication of cell-laden alginate microgels, a typical process involved (i) emulsification of cell-laden aqueous suspension in an immiscible, non-polar liquid (continuous phase) and (ii) the formation of droplets containing alginate precursor solution which can be subsequently gelled. Alginate microgels can be crosslinked by divalent ions such as calcium ions (Moyer et al., 2010; Yao et al., 2012; Pereira et al., 2013). The gelation of alginate relies on the crosslinking reaction that occurs rapidly between calcium ions and the α-L-guluronic (G) fragment of the polymer chain (Goh et al., 2012). Two gelation strategies of alginate microfluidic microgels are commonly adopted, i.e., external gelation in which crosslinkers are diffused outside of the droplets (Tan and Takeuchi, 2007; Zhang et al., 2007; Utech et al., 2015; Hati et al., 2016) and internal gelation in which crosslinkers are loaded within the aqueous phase and trigger-gellified upon stimuli (Huang et al., 2006). The external approach was widely used for the preparation of alginate beads with a size ranging from several hundreds of microns to millimeters. These methods were reported to lead to an exposure of cells to potential cytotoxicity of a high concentration of Ca^2+^ and compromised injectability due to their large size (Kang et al., 2014). Instead, the method of internal gelation can be triggered by the release of Ca^2+^ ions upon the diffusion of acetic acid from the continuous phase into the aqueous phase using calcium sources such as CaCO_3_ (Tan and Takeuchi, 2007) or CaSO_4_ (Kong, 2003) particles. This method, however, suffers from problematic issues such as inhomogeneous hydrogel network formation and high cytotoxicity upon prolonged exposure to surfactants or crosslinkers (Tan and Takeuchi, 2007; Zhang et al., 2007; Choi et al., 2016). To overcome these problems, the introduction of soluble calcium complexes was recently developed as a more promising strategy for rapid and uniform gelation upon Ca^2+^ ion release triggered by the acid. For instance, calcium chelated with ethylenediaminetetraacetic acid (EDTA) to form a calcium complex was used as a crosslinker for alginate to enable the release of Ca^2+^ ions at an acidic environment (pH < 5.0) (Utech et al., 2015). Since alginate and Ca-EDTA are both water soluble, Ca^2+^ ions can be uniformly dispersed in the alginate precursor solution to allow further internal gelation to form a uniform hydrogel network. However, the cell viability and activities can be substantially compromised due to extensive exposure to the acidic condition during the fabrication process (more than a few minutes) (Utech et al., 2015). To solve the problem, Hati et al. (2016) proposed a method of on-chip triggered gelation at pH 6.7. Zn^2+^ ions are able to exchange between ethylenediamine-*N*,*N*’-diacetic acid (EDDA) and EDTA due to the difference in dissociation constant, thus resulting in the release of Ca^2+^ ions at a relatively neutral pH (Hati et al., 2016). It is promising to exploit calcium complexes with a high equilibrium dissociation constant for precisely controlled on-chip gelation and high cell viability by introducing a rather mild crosslinking condition for cell encapsulation. With this aim, a comprehensive evaluation of different calcium complexes as crosslinking agents for the microfluidic preparation of cell-laden alginate microgels is desired.

Herein, we reported a biocompatible microfluidic approach for on-chip gelation of alginate microgels using calcium complexes that can be released in a more physiologically relevant condition. We introduced nitrilotriacetic calcium (Ca-NTA) with a relatively high dissociation constant as the calcium source and compared it with Ca-EDTA by evaluating the dissociation energy and gelation time for on-chip microgel formation. We further demonstrated the viability of encapsulated fibroblasts and the biofunctionality of encapsulated MSCs using an osteogenic differentiation model.

## Materials and Methods

### Isothermal Titration Calorimetry (ITC)

To evaluate the dissociation constant of calcium complexes, an ITC (MicroCal iTC200, United States) method was used as previously reported (Rafols et al., 2016). Specifically, calcium chloride (CaCl_2_, Sigma, United States) solution was titrated into the EDTA or NTA solution which was buffered to a defined pH (pH 6.5 and pH 7) using 4-morpholineethanesulfonic acid hydrate (MES, Solarbio, China) or *N*-2-hydroxyethylpiperazine-*N*-2-ethane sulfonic acid (HEPES, Solarbio, China). Both CaCl_2_ and EDTA (Sigma, United States) were configured using buffers. The concentrations of CaCl_2_ and EDTA/NTA were 10^–2^ and 10^–3^ M, respectively. All solutions were degassed before the test. To carry out the titration, the syringe was loaded with the CaCl_2_ solution, and the sample cuvette was filled with EDTA (or NTA) solution. The identical CaCl_2_ solution was used in the buffer as background titration to determine the dilution heat which was subtracted from the sample value. The solution in the cuvette was stirred at 1,000 rpm by the syringe to ensure a rapid mixing. Typically, 2 μl of titrants was injected into a known volume of samples placed in the cuvette during 60 s. The number of additions was 19 with an adequate interval of 150 s between injections to allow complete equilibrations. All ITC titrations were carried out at 37°C.

### Cell Isolation and Culture

Fibroblasts (NIH3T3, ATCC, China) were cultured in DMEM (HyClone, United States) with 10% of fetal bovine serum (FBS, Gibco, United States) and 1% of penicillin/streptomycin at 37°C and 5% CO_2_. Primary rat bone marrow-derived MSCs were isolated from 3-week-old male Sprague-Dawley (SD) rats. In brief, cells were flushed out of the marrow cavity and sieved through a 70-μm-mesh strainer and then collected by centrifugation (300 *g* for 5 min). Cells were then cultured in α-Minimum Essential Medium (α-MEM) with 10% of FBS and 1% of penicillin/streptomycin (Gibco, United States). Non-adherent cells were removed after 36 h before fresh medium was added. MSCs were cultured and passaged when cells reached 80% confluency, and MSCs at passages of 3–5 were collected for further experiments.

### Fabrication of PDMS Microfluidic Devices

PDMS microfluidic devices were fabricated by soft lithography protocol (Qin et al., 2010). In brief, a negative photoresist SU-8 (MicroChem, United States) was spin-coated onto a clean silicon wafer to a thickness of 50 μm and baked at 65°C for 3 min and then 95°C for 8 min. Subsequently, through a transparency photomask (Newway, China) designed by CAD (Art Service, United States), an exposure energy of 200 mJ/cm^2^ was used for UV exposure. Then the postexposure bake was carried out at 65°C for 1.5 min and further at 95°C for 6 min. To develop the microstructure, the sample was immersed in a photoresist developer for 5 min and washed three times by isopropyl alcohol. All chips were then dried with filtered nitrogen before being baked for 2 min at 150°C to ensure the stability of SU-8. After the microstructure fabrication, poly(dimethylsiloxane) (PDMS) and crosslinker (base/crosslinker = 10/1) were poured onto the pattern and solidified overnight at 85°C. PDMS molds were peeled off the silicon plate, and channel inlets and outlets were made by a biopsy punch of 1-mm diameter (Wenhan, China). The obtained PDMS replica was bonded to a glass slide after oxygen-plasma treatment and cured for 1 h at 85°C. To render the channel surface hydrophobic, all chips were treated with the Aquapel (a hydrophobic silane, PPG Industries, Pittsburgh, PA, United States). All microfluidic chips were finally dried by the filtered nitrogen before being baked at 85°C.

### Microfluidics Generation of Alginate Microgels

A homogeneous hydrogel precursor solution containing 1% (w/v) alginate and 50 mM calcium complex was prepared. CaCl_2_ solution and Na-EDTA (Na-NTA) solution were mixed at a 1:1 molar ratio to obtain Ca-EDTA (Ca-NTA) solution, followed by the neutralization to pH 7.4 using sodium hydroxide (potassium hydroxide solution). Fluorinated carbon oil (HFE7100, 3M, United States) containing 0.5 v/v% Krytox-polyethylene glycol (PEG)-Krytox surfactants (DragonDrop, China) and acetic acid (0.05–1 v/v‰) was used as the continuous phase, and HFE7100 containing 20 v/v% PFO (Aladdin, China) was used as the washing phase to disturb the stability of the water/oil interface for de-emulsification. All liquids were separately added into syringes for injection into the chip. Polyethylene tubes with an inner diameter of 0.38 μm were used to connect the syringes and the PDMS devices. The flow rates were individually controlled by syringe pumps. The flow rate of the aqueous phase was kept constant at 100 μl/h to avoid cell damage from the shearing force during the injection, and the flow rates of the continuous phase and PFO oil phase were both set at 1,000 μl/h.

The gelation time of microgels was determined by the crosslink density of the hydrogel. To calculate the gelation time of microgels in the microfluidics channel, microfluidic chips with channels of different lengths were fabricated to estimate the gelation time of microgel within the chip. Specifically, all microfluidic chips contained two flow-focusing junctions which were connected using a straight microchannel of different lengths (between 1 mm and 4 cm). Droplets containing alginate precursor were generated by the first flow-focusing junction and were trigger-gellified within the downstream straight channel where acetic acid from the oil phase diffuses into the aqueous droplets to initiate the gelation process. Upon converging into the second flow-focusing junction, a buffering phase containing 25 mM HEPES (pH 7.4) to neutralize the pH was introduced; this will quench the crosslinking reaction. Here, the flow rate of the aqueous phase was kept constant at 100 μl/h, while those of the oil phase and washing phase were set at 1,000 μl/h. If the in-drop gelation of alginate can be finalized, spherical solid-like hydrogel particles can be collected from the outputting solution. Otherwise, no spherical microparticles or even no microparticles can be collected since the time for the droplets to finalize alginate crosslinking was not enough. We denoted the latter phenomenon as “uncrosslinked droplets.” The morphology of the resulting microgels characterized by different degrees of roundness further provided quantitative information of the crosslinking degree. For the resulting microgels with a high degree of roundness, we considered them as “completely crosslinked microgels.” In contrast, microgels showing poor roundness or particle aggregation can be considered as “incompletely crosslinked microgels.” Subsequently, the real-time detection of microgel flow was collected by a high-speed camera (i-SPEED 221, iX Cameras, United Kingdom), and the flow time of completely crosslinked microgels can be determined.

### Microfluidic Generation of Cell-Laden Microgels

Arg-Gly-Asp (RGD)-modified sodium alginate was synthesized by the carbodiimide chemistry according to our published work (An et al., 2020). In brief, 1 g sodium alginate (BioReagent, Sigma, United States) was completely dissolved in 100 ml MES buffer solution (0.1 M MES, 0.3 M NaCl, pH 6.5). Then, sequentially added into the sodium alginate solution were 48.42 mg of EDC (Aladdin, China), 27.4 mg of sulfo-NHS (Aladdin, China), and 16.7 mg of peptide motif G4RGDY (Wuhan Holder, China), which were reacted at least 20 h with stirring and finally stopped by adding 18 mg of hydroxylamine hydrochloride. To obtain purified RGD-alginate, the resulting solution was dialyzed with a dialysis tube (MWCO 3500, Solarbio, China) over 3 days. Afterward, the dialyzed solution was sterilized by a 0.22-μm filter and then lyophilized to obtain sodium alginate powder.

Cells were dispersed in alginate solution with a final cell density of 3 × 10^6^ cells/ml in 1 w/v% alginate and 50 mM Ca-EDTA/Ca-NTA. The composition of the continuous phase and the aqueous phase was described above, but 0.4 and 0.2 v/v‰ acetic acid were used in the Ca-EDTA system and Ca-NTA system, respectively. The flow rate of the aqueous phase was kept constant at 100 μl/h, and the flow rates of the continuous phase and PFO were both set at 1,000 μl/h. The collection solution to retreat the encapsulated cells was composed of α-MEM plus 25 mM HEPES (pH 7.4) to neutralize the pH of the aqueous phase. The cell-laden microgels were finally collected by centrifugation (300 *g* for 5 min) before being redispersed in the medium.

### Cell Viability

To assess the biocompatibility of the encapsulation process, 3 × 10^6^ fibroblasts were mixed with 1 ml alginate precursor solution and kept in the syringe for 1 and 4 h, respectively. Fibroblasts were subsequently collected and washed three times with PBS to remove the alginate. The remaining fibroblasts were centrifuged (300 *g* for 5 min) and counted in the tissue culture plates. To evaluate the biocompatibility of produced alginate microgels, cell-laden microgels were also collected by centrifuge (300 *g* for 5 min) after 1 day of culture and seeded in tissue culture plates. Fibroblast were further stained with 2 mM calcein-AM (Invitrogen, China) and 4 mM ethidium homodimer (Invitrogen, China) for 15 min at room temperature. Samples were then observed using a confocal laser scanning microscope (OLYMPUS FV3000, Japan), and the cell viability was analyzed.

### Cell Metabolism Activity

Cell metabolism activity was determined using a CCK-8 assay (Yisheng, China) according to the instruction of the manufacturer. In brief, cell-laden microgels were collected by centrifugation (300 *g* for 5 min) after 1 day of culture, and then 1 ml of CCK-8 work solution was added and incubated for 2 h at 37°C. After that, cell-laden microgels were centrifuged, and 200 μl of supernatant was transferred into 96-well plates. The absorbance at 450 nm was measured using a microplate reader (Bio-Rad iMark, United States). The final values of each group were determined by subtracting the value of the blank control.

### Nuclear Staining

Nuclear staining of encapsulated cells was determined according to the instruction of a SYTO 9 green-fluorescent nucleic acid kit (Invitrogen, China). In brief, microgels were collected by centrifugation (300 *g* for 5 min) after 1, 4, 7, 14, and 21 days of culture, and the medium was refreshed. SYTO 9 dyes of 5 μM were subsequently added and incubated for 10 min at room temperature before being observed using a confocal laser scanning microscope (OLYMPUS FV3000, Japan).

### Differentiation of Stem Cells in Microgels

Into six-well non-adherent plates were placed 1 × 10^5^ MSC-laden microgels (NEST, China). After 3 days of culture in the growth medium, MSC-laden microgels were cultured in the osteogenic medium (α-MEM supplemented with 100 nM dexamethasone (Sigma, United States), 10 mM β-glycerophosphate (Sigma, United States), and 50 μM ascorbic acid (Sinopharm, China), and the medium was refreshed every 3 days. After 1, 4, 7, 14, and 21 days, microgels were collected and digested with 500 μl of EDTA solution at 37°C until the microgels were completely degraded. Afterward, samples were centrifuged (300 *g* for 5 min) and washed three times with PBS and then suspended in 250 μl of RIPA lysis buffer (Solarbio, China) for 15 min. The DNA content of encapsulated cells was quantified using the PicoGreen dsDNA Quantitation Reagent (Yisheng, China) per the manufacturer’s instruction. A 4-nitrophenyl phosphate-based method was applied to assess the alkaline phosphatase (ALP) activity of encapsulated cells according to the instruction of an Alkaline Phosphatase Assay Kit (Beyotime, China). ALP activity was further normalized to the dsDNA content.

### Mineralized of MSC-Laden Microgels

The mineralization of microgels was quantified by the opacity of microgels. In brief, after 1, 4, 7, 14, and 21 days of culture, cell-laden microgels were observed with a phase contrast microscope (Motic AE2000, China), and the gray value of microgels was evaluated using ImageJ. The mineralization of cell-laden alginate microgels was also characterized with the Alizarin Red S staining (Solarbio, China). In brief, after 21 days of culture in the osteogenic medium, microgels were collected and washed with PBS and then fixed with 4% PFA for 15 min; microgels were then stained with 0.1% Alizarin Red S for 15 min before being observed with the phase contrast microscope.

### Statistical Analysis

All data are presented as means ± SD. To determine the significant difference, comparisons between Ca-NTA and Ca-EDTA were performed by Student’s *t*-test, and other comparisons between different groups were performed by a one-way ANOVA with a Tukey *post-hoc* multiple comparison. Levels of statistical significance were set at ^∗^*p* < 0.05, ^∗∗^*p* < 0.01, and ^∗∗∗^*p* < 0.001.

## Results and Discussion

### Formation of On-Chip Gelation in Microfluidic Droplets

**FIGURE 1:**
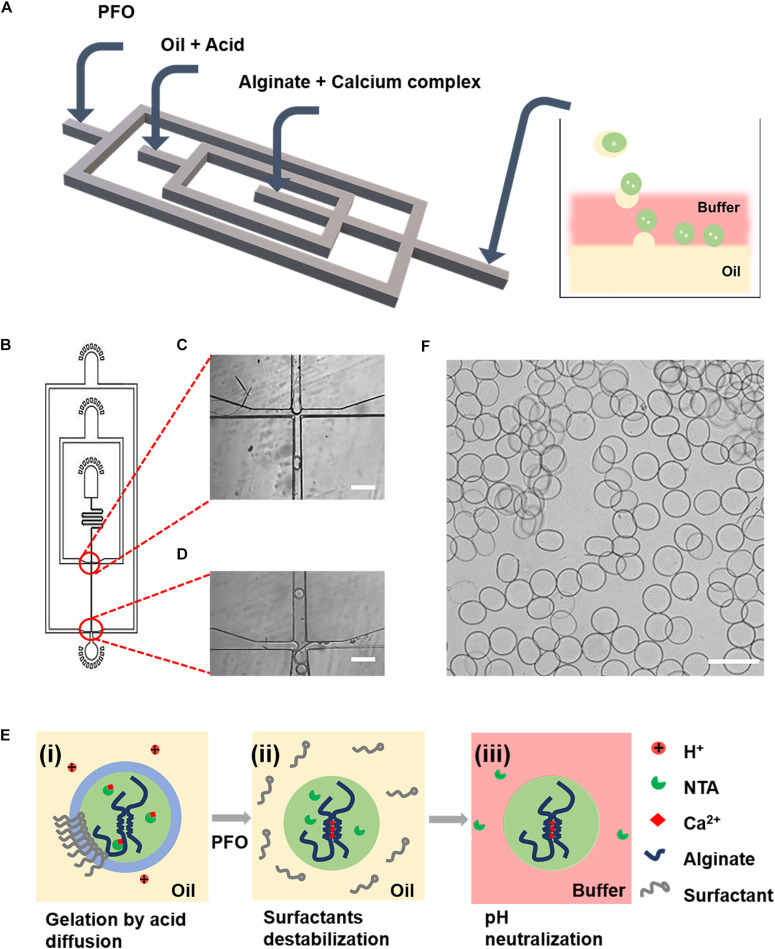
Microfluidic generation of alginate gelation by on-chip release of calcium ions from a water-soluble calcium complex as the crosslinker. **(A)** Schematic illustration of the design of the PDMS device for the generation of cell-laden alginate microgels. **(B)** AutoCAD design of the microfluidic device for continuous generation of cell-laden microgels. **(C,D)** Microscopic images of the microchannels showing the flow-focusing drop-maker **(C)** and the cross-junction for adding of PFO **(D)**. **(E)** Schematics showing the formation mechanism of alginate microgels. (i) Flow-focusing junction breaking up the lamellar flow into droplets. The acetic acid in the oil phase diffused into the aqueous droplet, which triggered the release of Ca^2+^ ions from calcium complex conjugates and induced the gelation of alginate. (ii) The stability of the oil–water interface was broken by PFO. (iii) Microgels were immediately transferred from the oil to an aqueous phase consisting the buffer until the pH of microgels was neutralized. **(F)** Microscopic image showing the resulting alginate microgels. Scale bar is 100 μm.

To generate alginate microgels, a microfluidic device (Figures 1A,B) integrating functions of droplet formation (Figure 1C), in-drop gelation, and de-emulsification (Figure 1D) was used (Zhang et al., 2018; An et al., 2020). To trigger the gelation of alginate, we used calcium complexes, i.e., Ca-EDTA and Ca-NTA, as the crosslinker, which can chelate with calcium ions and remain soluble in the aqueous phase without reacting with alginate molecules. After the formation of droplets, alginate gelation was induced by the addition of acetic acid in the oil phase (Figure 1Ei). The stability of the oil–water interface was broken by the injected PFO through an additional channel (Figure 1Eii). Microgels were then immediately transferred from the oil to an aqueous phase consisting of the buffer until the pH of microgels was neutralized (Figure 1Eiii). Microgels were finally collected from the water–oil mixture solution by centrifugation (Figure 1F). Although a previous study has shown that Ca-EDTA as a calcium source for on-chip gelation can enable continuous production of cell-laden microgels without significantly compromising cell viability (Hati et al., 2016); the detrimental effects on the encapsulated cells of a high concentration of acid to dissociate Ca-EDTA and the long-term cell functionality have not been proved.

**FIGURE 2:**
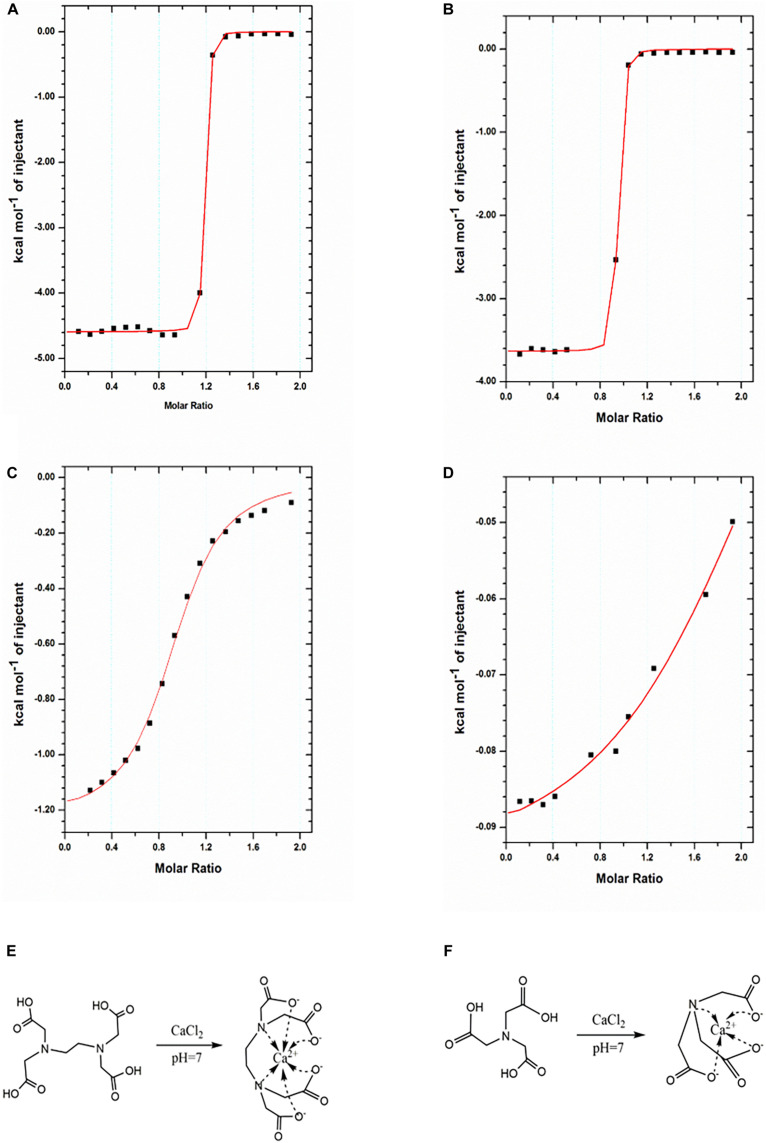
Characterization of binding interactions by ITC. **(A,B)** ITC titration of EDTA (1 mM) with Ca^2+^ ions (10 mM) at 25°C in pH 7 **(A)** and pH 6.5 **(B)**. **(C,D)** NTA (1 mM) with Ca^2+^ ions (10 mM) at 25°C in pH 7 **(C)** and pH 6.5 **(D)**. The reaction schemes for the formation of the Ca-EDTA complex **(E)** and Ca-NTA complex **(F)**.

Aiming at this, here, we hypothesized that the replacement of Ca-EDTA by Ca-NTA may lead to enhanced biocompatibility of the microfluidic fabrication process since the triggered release of Ca^2+^ ions from NTA can be achieved by less acid due to the lower chelation energy between Ca^2+^ ions and NTA relative to EDTA. To prove this, we compared the equilibrium dissociation constant between Ca^2+^ ions and EDTA vs. NTA molecules using ITC. The ITC measurements monitored the heat change during the titration of EDTA (or NTA) solution with CaCl_2_ solution at different pH values. Typically, a plot of heat change dependent on the molar ratio of CaCl_2_ to EDTA (or NTA) can be obtained (Figure 2). For compounds with strong reciprocal affinity, the graph typically showed a sharp variation of heat upon the concentration of the titrant until the reaction toward saturation. The molar ratio at the center of the binding isotherm indicated the reaction stoichiometry (N) and the number of binding sites (Rafols et al., 2016). Herein, we observed that the stoichiometry of both Ca-EDTA and Ca-NTA upon the molar ratio of Ca^2+^ ions to EDTA (or NTA) molecules reached 1:1 (Table 1). The slope of the linear region of the sharp increase in the calorimetric curve corresponded with K_*ITC*_ values which represented the dissociation constant. Therefore, a lower K_*ITC*_ value corresponded with a higher dissociation constant, indicating that the components tend to be dissociated. As shown in Table 1, the K_*ITC*_ value of Ca-NTA (or Ca-EDTA) declined with pH decrease, as evidenced by the K_*ITC*_ value of 1.95 ± 0.224 × 10^4^ M^–1^ at pH 7 decreasing to 8.66 ± 1.96 × 10^6^ M^–1^ at pH 6.5. This indicated that lowering the pH can ease the dissociation of the calcium complex and trigger the release of Ca^2+^ ions. Moreover, in the comparison of Ca-NTA and Ca-EDTA, the former combination showed lower K_*ITC*_ value and thus higher dissociation constant, which suggested that Ca-NTA was more likely to be dissociated at the same pH. These findings confirmed our hypothesis that the release of Ca^2+^ ions can be triggered using less acid from Ca-NTA rather than Ca-EDTA.

**TABLE 1:** Ca^2+^-EDTA/NTA binding parameters at 25°C.

	pH	*N*	K_*ITC*_ (M^–1^)
Ca-EDTA	7	1.14 ± 0.00174	(8.66 ± 1.96) × 10^6^
Ca-EDTA	6.5	0.908 ± 0.00153	(3.48 ± 0.646) × 10^6^
Ca-NTA	7	0.927 ± 0.00122	(1.95 ± 0.224) × 10^4^
Ca-NTA	6.5	2.24 ± 0.0792	(5.49 ± 1.35) × 10^3^

### On-Chip Formation of Alginate Microgels

**FIGURE 3:**
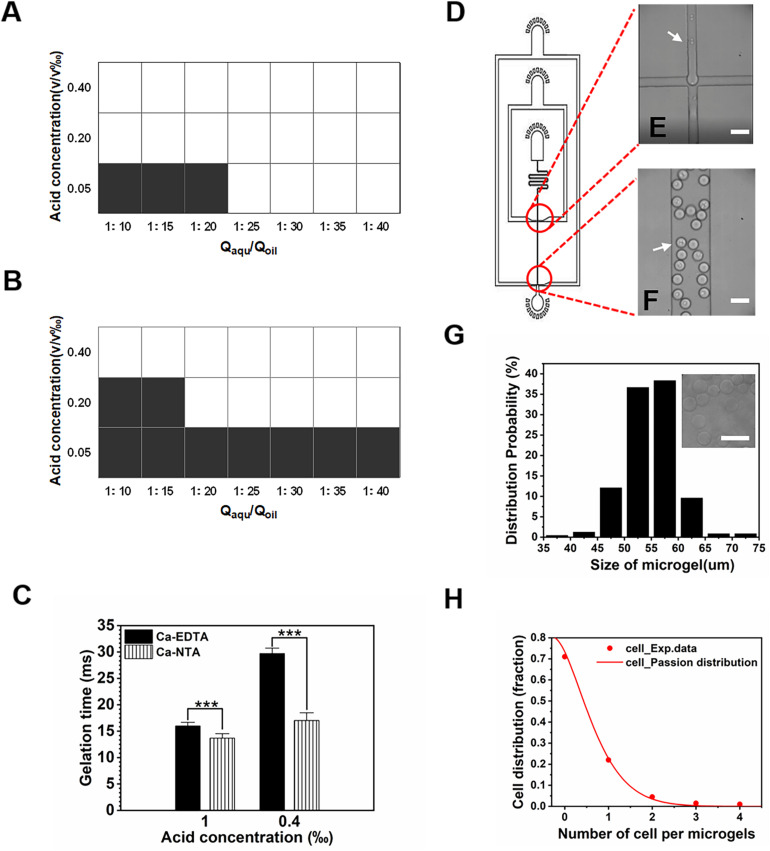
Gelation triggered by different calcium complexes. **(A,B)** Effect of water–oil flow rate ratio (Q_*aqu*_/Q_*oil*_) and acetic acid concentration on the formation of alginate microgels when **(A)** Ca-NTA and **(B)** Ca-EDTA were used as the crosslinker, respectively. White and black areas indicated complete crosslink and uncrosslink, respectively. If the gelation process can be achieved on-chip, solid-like hydrogel particles can be obtained. Otherwise, only liquid can be collected since the gelation of the droplets cannot be realized within the short time window, for which we considered them as uncrosslinked microgels. **(C)** Gelation time in different acid concentrations where Ca^2+^ ions were chelated in NTA or EDTA. **(D)** AutoCAD design of the microfluidic device for cell encapsulation. **(E,F)** Microscopic images of microchannels **(E)** for formation of alginate cell-laden microgels **(F)**. White arrows indicate cells. **(G,H)** Effect of Ca-NTA on microgel size and cell distribution. **(G)** Size distribution of alginate microgels. **(H)** Cell distribution in the microgels follows the Poisson distribution. Scale bar is 100 μm (***p < 0.001).

We further investigated the influence of two crosslinkers (i.e., Ca-EDTA vs. Ca-NTA) on the fabrication process of alginate microgels, including the concentration of acetic acid to trigger-release Ca^2+^ ions, the on-chip gelation time, and the flow rates of each inputting liquid. We observed that the threshold concentration to trigger the on-chip release of Ca^2+^ ions from Ca-NTA at a fixed aqueous/oil flow rate ratio Q_*aqu*_/Q_*oil*_ = 1/10 (Q_*aqu*_ = 100 μl/h) was 0.2 v/v‰ (Figure 3A), while the threshold concentration of acetic acid for Ca-EDTA was 0.4 v/v‰ (Figure 3B). Apparently, the introduction of Ca-NTA as the crosslinker requested less acetic acid for triggering the release of Ca^2+^ ions compared to Ca-EDTA. Moreover, the increase of flow rate ratio Q_*aqu*_/Q_*oil*_ eased the formation of alginate microgels. For instance, alginate microgels can be formed at a considerably low acetic acid concentration of 0.05 v/v‰ by using Ca-NTA as the crosslinker (Q_*aqu*_/Q_*oil*_ = 1:25). This might be attributed to the accumulation of acetic acid dissolved in the oil phase, which enhanced the total amount of acid accessible to the aqueous droplets upon increasing Q_*aqu*_/Q_*oil*_ values. In sum, these results suggested that the use of Ca-NTA instead of Ca-EDTA can ease the preparation of alginate microgels by reducing the desired dose of acetic acid.

The gelation time was important for cell viability (Utech et al., 2015; Hati et al., 2016), fluid stability (Huang et al., 2006), and chip design. We further investigated the time needed to completely gellify alginate droplets. By adjusting the length of the downstream channel after the flow-focusing drop-maker, we can determine the desired minimum gelation time by assessing different morphologies of collected microgels. For chips designed with a rather long downstream channel, alginate microgels with desirable spherical morphology without aggregation can be obtained. By reducing the length of the downstream channel, we can evaluate the morphology of resulting microgels and confirm the threshold gelation time if spherical alginate particles cannot be obtained. We found that Ca-NTA showed a significantly shorter gelation time of 13.67 ± 0.85 ms upon using 1 v/v‰ acetic acid to trigger alginate gelation compared to Ca-EDTA (15.98 ± 0.70 ms) (Figure 3C). Upon lowering the acid concentration to 0.4 v/v‰, the gelation time of alginate triggered by Ca-NTA increased to 17.01 ± 1.48 ms compared to that of Ca-EDTA (29.71 ± 1.03 ms) (Figure 3C). A previous study by Hati et al. (2016) measured the gelation time of alginate hydrogels using a rheometer; it showed that the gelation time for 3.5 ml of alginate hydrogels was 9.0 ± 3.5 s using Zn^2+^ exchange between EDDA and EDTA to trigger gelation at a relatively mild condition. The gelation time reported here was more than two magnitudes shorter than their microfluidic strategy, which can be attributed to the lower diffusion efficiency measured using a rheometer. Therefore, the present method by using Ca-NTA as a crosslinker considerably reduced the acid concentration needed to trigger Ca^2+^ release but also significantly decreased the gelation time, which can ease the design of microfluidic chip for continuous generation of cell-laden microgels.

We further characterized these cell-laden microgels using the present approach. The cell-laden alginate/Ca-NTA mixture was emulsified into monodisperse droplets (Figure 3D). With the use of the chip with a 50-μm channel (Figure 3E) and acid concentration of 0.2 v/v‰, cell-laden microgels (Figure 3F) ranging in size from 50 to 60 μm were produced (Figure 3G). We observed that the distribution of cell numbers in each microgels followed the Poisson distribution (Figure 3H) and met with the results from previous studies using microfluidic droplet-based strategies (Koster et al., 2008; An et al., 2020). Of the microgels, 22% contained single cells, while the majority of the microgels (71%) remained empty, and a few droplets contained more than one cell (Figure 3H).

### Biocompatibility of Microfluidic Fabrication Process

**FIGURE 4:**
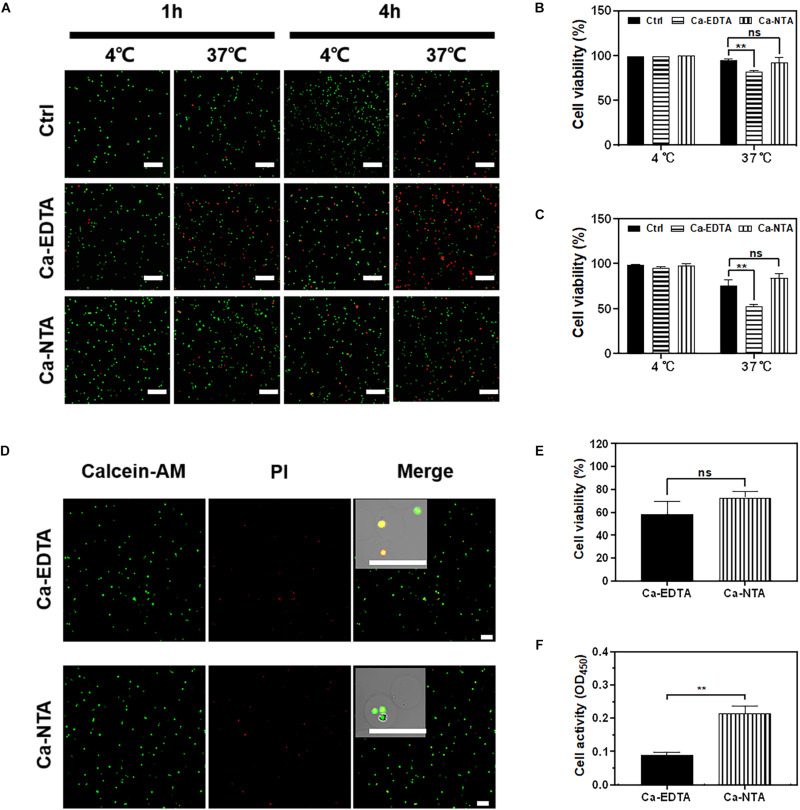
Evaluation of cell viability for microfluidic fabrication process. NIH3T3 fibroblasts were cultured in medium containing 1 wt% Na-alginate and 50 mM Ca-EDTA or Ca-NTA, incubated in a sealed syringe at 4°C and 37°C for 1 and 4 h, respectively. **(A)** The confocal microscopic images of live (green) and dead (red) cells stained by Calcein-AM and ethidium homodimer, respectively. The cells were incubated **(B)** 1 h and **(C)** 4 h. The numbers of live and dead cells were counted with the aid of ImageJ. The viability of NIH3T3 fibroblasts in microgels. To encapsulate cells, Ca-NTA and Ca-EDTA were used as the crosslinker. **(D)** Live (green) and dead (red) cells were stained by calcein-AM and ethidium homodimer, respectively. **(E)** The numbers of live and dead cells from microgels that were triggered by Ca-NTA or Ca-EDTA were counted with the aid of ImageJ. **(F)** Cell activity of encapsulated cells was assessed by CCK-8 assay after 24 h of culture. Scale bar is 200 μm (^∗∗^*p* < 0.01).

The biocompatibility of the microfluidic fabrication process is the prerequisite for applications of cell-loaded microgels. Therefore, the viability and metabolic activity of the cells after the fabrication process were assessed. In typical microfluidic processes for cell encapsulation, cells were mixed with hydrogel precursor solution for hours in a sealed syringe. Therefore, cell viability is frequently compromised due to the lack of nutrients and oxygen. We mimicked this process by mixing cells with alginate precursor and crosslinkers (Ca-EDTA or Ca-NTA) in a sealed syringe and stored at 4 and 37°C, respectively. The NIH3T3 fibroblasts were further observed, and their viability was analyzed after 1 and 4 h of maintenance in the syringe (Figures 4A–C). No significant difference in cell viability was observed at a low temperature (4°C) compared to the control group in which cells were suspended and maintained in the medium (Figures 4B,C). As a result, cells incubated with alginate precursor and Ca-NTA at 37°C for 1 h (92.33 ± 4.64% vs. 94.33 ± 1.70%) and 4 h (84.00 ± 3.74% vs. 75.67 ± 4.99%) showed no significant difference in cell viability compared to the control group (Figures 4B,C). However, cell viability of the Ca-EDTA group (81.67 ± 1.24%) was significantly declined compared to the control (94.33 ± 1.70%) after 1 h and more obviously reduced (52.00 ± 2.16% vs. 75.67 ± 4.99%) after 4 h. These results suggested that cells can be maintained at a low temperature (4°C) for high viability even for several hours. Also, the use of Ca-NTA as the crosslinker had superior biocompatibility than that of Ca-EDTA.

We further evaluated the biocompatibility of microgels produced using Ca-NTA vs. Ca-EDTA as the crosslinker and using different concentrations of the acid (0.2 v/v‰ for Ca-NTA and 0.4 v/v‰ for Ca-EDTA) in the continuous phase (Figures 4D–F). We did not observe a significant difference of cell viability prepared using Ca-NTA (72.75 ± 4.60%) and Ca-EDTA (58.25 ± 9.91%) after 1 day of encapsulation (Figure 4E). However, the metabolic activity using CCK-8 assay showed that cells prepared using Ca-NTA had twice as high metabolic activity as Ca-EDTA. This indicated that Ca-NTA can provide a more bio-friendly environment for cell encapsulation in alginate microgels.

### Viability and Functionality of the Encapsulated Cells

**FIGURE 5:**
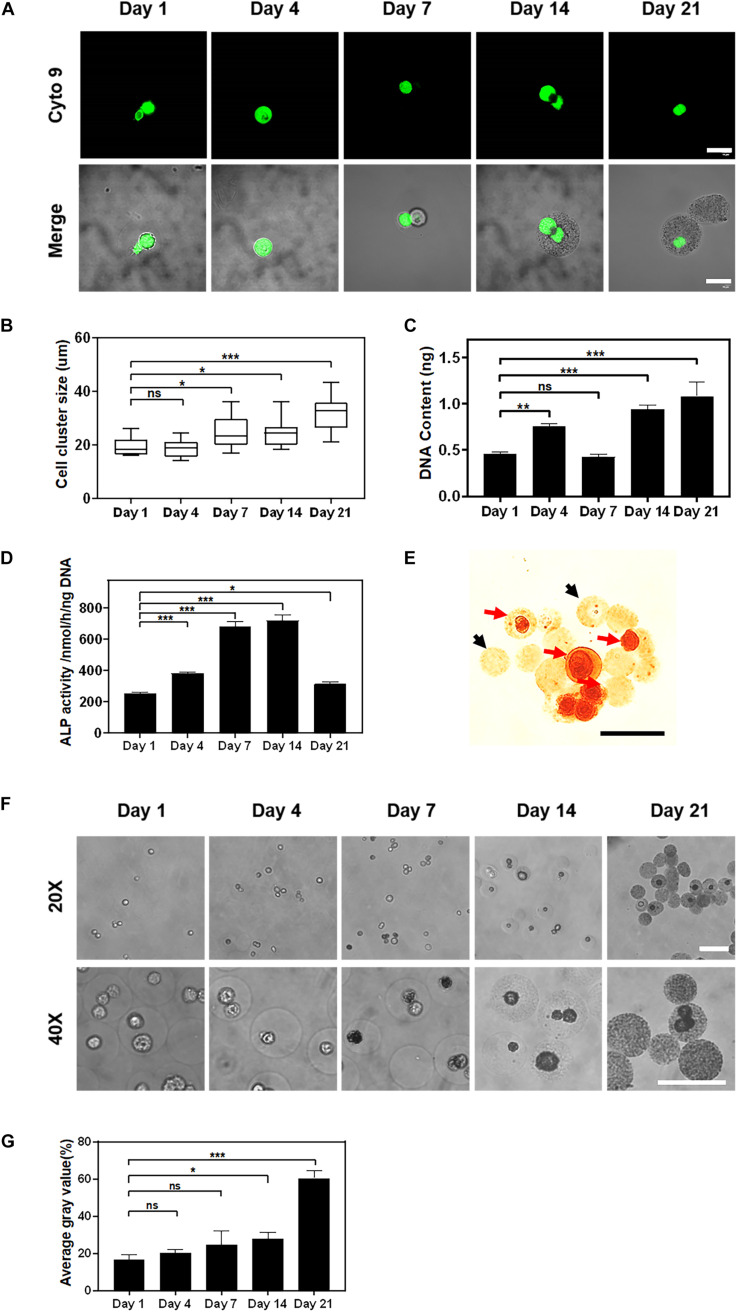
The proliferation and osteogenic differentiation of stem cells encapsulated in alginate using the Ca-NTA complex. **(A)** Confocal microscopic images of MSCs encapsulated in alginate microgel after SYTO 9 nucleus staining (green). Scale bar is 20 μm. **(B)** Quantify the average diameter of cell clusters within the microgels at different time points using ImageJ. **(C)** The cell proliferation was further determined by the DNA content. **(D)** Osteogenic differentiation of MSCs was measured by ALP activity at different time points. **(E)** Cell-laden microgels were stained with 1% Alizarin Red S. The black arrow indicated microgels without cells, and the red arrow indicated cell-laden microgels. **(F)** Light microscopic images of microgels under the phase contrast microscope, and **(G)** the mineralization of the cell-laden microgels was determined based on the average gray value of microgels. Scale bar is 100 μm (ns > 0.05, *p < 0.05, **p < 0.01, ***p < 0.001).

The biological functions of encapsulated cells play a key role in practical applications such as cell therapy and tissue regeneration. Here, we evaluated the biological functions of encapsulated cells by using an osteogenic differentiation model with rat MSCs since it is a typical regeneration model and allogeneic cells are planned to be encapsulated for tissue engineering for further animal study. When MSCs were encapsulated in alginate microgels using Ca-NTA as the crosslinker and cultured in the osteogenic medium (Figure 5A), the average diameter of cell clusters within the microgels significantly increased from 19.58 ± 3.50 to 32.20 ± 5.70 μm from 1 to 21 days (Figure 5B). These findings indicated a continuous cell division and cell proliferation in microgels. The cell proliferation was further determined by the DNA content. It showed that MSCs proliferated from 1 day (0.46 ± 0.02 ng) to 21 days (1.08 ± 0.12 ng) (Figure 5C).

The differentiation of MSCs was further assessed by the ALP activity and the mineralization of encapsulated MSCs. As a potential maker of osteogenic differentiation of MSCs, the ALP activity of encapsulated MSCs increased rapidly after 7 days (684.80 ± 23.40 nmol/h/ng) of *in vitro* culture and peaked by 14 days (721.14 ± 29.54 nmol/h/ng) followed by a sharp decrease afterward (314.07 ± 11.50 nmol/h/ng) (Figure 5D). This finding suggested that MSCs encapsulated in alginate microgels underwent the osteogenesis process. The reduction of ALP activity at a later time point was presumed to be due to the mineralization of MSCs as previously reported (Zhang et al., 2019). Further Alizarin Red S staining for calcium deposition in the hydrogel matrix indicated the distribution and formation of calcium-based minerals. To distinguish the mineralization from introduced calcium ions, we used empty microgels as a negative control. The results showed that empty microgels were stained faint yellow while cells in microgels were stained scarlet (Figure 5E). This suggested that the mineralization of the microgel matrix was attributed to the osteogenic differentiation of encapsulated MSCs. We further characterized the mineralization process by monitoring the change in opacity of the cell-laden microgels. We observed that cells and microgels transformed from transparent to opaque after 7 days of osteogenic culture (Figure 5F). Quantitatively, the degree of opacity did not show a significant difference between 1 day (16.44 ± 2.41%) and 7 days (24.48 ± 6.15%) of culture but exhibited a substantial increase as evidenced by the increase of gray values from 24.48 ± 6.15% at day 7 to 60.40 ± 3.25% at day 21 of culture (Figure 5G). To sum up, these findings suggested that MSCs encapsulated in alginate microgels maintained their functionality and committed osteogenic differentiation upon inductive culture. Therefore, the current approach for the preparation of cell-laden alginate microgels by using Ca-NTA as the crosslinker is able to maintain high viability and the long-term functionality of encapsulated cells.

## Conclusion

We hereby presented an effective and biocompatible method for encapsulating single cells using alginate microgels. The efficiency of triggering the release of calcium ions using Ca-EDTA vs. Ca-NTA was evaluated by characterizing the on-chip gelation time and pH and the equilibrium dissociation constant of both calcium complexes. It demonstrated that Ca-NTA had a higher dissociation constant compared to Ca-EDTA due to the release of calcium ions at a rather neutral pH. Compared to Ca-EDTA, Ca-NTA used as a crosslinker showed significantly higher cell viability and cell metabolic activity for encapsulated cells after the microfluidic process, as well as long-term functionality of encapsulated MSCs evidenced by the osteogenic differentiation model. This biocompatible strategy for cell encapsulation using microfluidic chips offers a powerful tool for future applications of alginate microgels in tissue engineering and cell-based therapies.

## Data Availability Statement

All datasets generated for this study are included in the article/supplementary material.

## Ethics Statement

The animal study was reviewed and approved by the Biology and Medical Ethics Committee, Dalian University of Technology.

## Author Contributions

FS, LY, and HW designed the study. FS and LY performed the experiment and collated the data. CA, HZ, YX, and YuZ helped with the experiments. FS, YaZ, and HW analyzed the data and wrote the manuscript. All authors contributed to the article and approved the submitted version.

## Conflict of Interest

The authors declare that the research was conducted in the absence of any commercial or financial relationships that could be construed as a potential conflict of interest.
